# Osmotic stress-induced somatic embryo maturation of coffee *Coffea arabica* L., shoot and root apical meristems development and robustness

**DOI:** 10.1038/s41598-021-88834-z

**Published:** 2021-05-06

**Authors:** Eliana Valencia-Lozano, Jorge E. Ibarra, Humberto Herrera-Ubaldo, Stefan De Folter, José L. Cabrera-Ponce

**Affiliations:** 1 Departamento de Biotecnología y Bioquímica, Centro de Investigación y Estudios Avanzados del IPN (CINVESTAV), Unidad Irapuato, Km. 9.6 Libramiento Norte Carretera Irapuato-León, CP 36824 Irapuato, Guanajuato Mexico; 2 Departamento de Ingeniería Genética, Centro de Investigación y Estudios Avanzados del IPN (CINVESTAV), Unidad Irapuato, Km. 9.6 Libramiento Norte Carretera Irapuato-León, CP 36824 Irapuato, Guanajuato Mexico; 3 Langebio. Unidad de Genómica Avanzada (CINVESTAV, UGA-LANGEBIO), Km. 9.6 Libramiento Norte Carretera Irapuato-León, CP 36824 Irapuato, Guanajuato Mexico

**Keywords:** Biotechnology, Molecular biology, Plant sciences

## Abstract

Somatic embryogenesis (SE) is the most important plant biotechnology process for plant regeneration, propagation, genetic transformation and genome editing of coffee, *Coffea arabica* L. Somatic embryo (SEs) conversion to plantlets is the principal bottleneck for basic and applied use of this process. In this study we focus on the maturation of SEs of *C. arabica* var. Typica. SEs conversion to plantlet up to 95.9% was achieved under osmotic stress, using 9 g/L gelrite, as compared with only 39.34% in non-osmotic stress. Mature SEs induced in osmotic stress developed shoot and root apical meristems, while untreated SEs were unable to do it. *C. arabica* regenerated plants from osmotic stress were robust, with higher leaf and root area and internode length. To understand a possible regulatory mechanism, gene expression of key genes of *C. arabica*, homologous to sequences in the *Arabidopsis thaliana* genome, were analyzed. A set of two component system and cytokinin signaling-related coding genes (*AHK1, AHK3, AHP4* and *ARR1*) which interact with *WUSCHEL* and *WOX5* homedomains and morphogenic genes, *BABY-BOOM, LEC1, FUS3* and *AGL15*, underwent significant changes during maturation of SEs of *C. arabica* var. Typica. This protocol is currently being applied in genetic transformation with high rate of success.

## Introduction

Coffee (*Coffea arabica* L. and *C. canephora* Pierre) is the most valuable tropical export crop worldwide, with an annual retail value of approximately US$ 88 billion. Its prices have increased by 160% during the last years**.** Like other fruit species, the generation of new varieties in coffee is time-consuming. In this scenario, the coffee culture improvement progress has been achieved mainly by plant breeding, which is time-consuming taking at least 20 years to have a new genotype in the market^1^. Somatic embryogenesis (SE) is one of the most widely applied plant biotechnology process for plant regeneration, micropropagation and genetic improvement by stable genetic transformation and genome editing. The complete plant regeneration of *C. arabica* derived from indirect SE was first achieved by Yasuda et al.^2^ using the cytokinin (BAP) as plant growth regulator. After that report, many approaches and protocols using the synthetic auxin (2,4-D) as an inducer of indirect SE have been tested with different rates of efficiency (For reviews, see:^3–9^).

Indirect SE is relatively well adapted to semi-industrial level using bioreactors^3^, although a technical constraint derived from the low embryo to plantlet conversion rate (30–50%), a long acclimatization stage (5–8 months) and asynchronicity still affect the whole procedure. Recently new bioreactor devices and the development of rooted mini-cuttings have improved the plantlet conversion rate^1,6^. One common problem related to SE, especially in woody tress is the low conversion of SEs to plantlet, as a result of incomplete maturation. Conversion is defined as root and epicotyl growth with new leaf development^10^. This is most likely due to a poor shoot and root apical meristem (SAM and RAM, respectively) development or defects in the meristem organization during SE. A proper understanding of apical meristem development and physiology will greatly enhance our ability to produce SEs of improved quality^11^.

SE maturation is a complex process that is influenced by hormone signaling pathways including abscisic acid (ABA), which is related to stress, and osmotic water potential of the medium. SEs are known to be stimulated to develop and mature in in vitro culture while environmental stresses are imposed, such as heat, nutrient depletion, solute-based water stress or increased levels of the plant hormone abscisic acid (ABA), whether added exogenously or induced endogenously^12^. Ontogeny of SEs of *C. arabica* was first demonstrated by Quiroz-Figueroa et al*.*^13^ from one single cell to embryo development. This group demonstrated that both, direct and indirect SEs have a unicellular origin. Etienne et al.^3^, analyzed developmental ontogeny of somatic versus zygotic embryos. They found morphological differences and lack of protein reserves in SEs at the beginning in the germination phase and marked differences in water characteristics compared to zygotic embryos (water potential, water content and relative water content). Histological evidences demonstrated that well developed shoot and root apical meristems (SAM and RAM respectively) in SEs were not induced. As noted previously by several reports, auxins negatively affect SE development in coffee^14–16^. Cytokinin signaling has demonstrated that plays a critical role during root and stem cell niche establishment allowing the RAM system initiation in SEs^17,18^. It also crosstalks cytokinins, ABA, ethylene, light, stress, MAPK cascade and glucose signaling. In this work we describe SEs maturation in *C. arabica* var. Typica, under osmotic stress and cytokinins. In the presence of high content of gelrite, mature SEs developed SAM and RAM demonstrated by histological analysis and were able of embryo to plantlet conversion and produce robustness plants compared to untreated SEs. We detected important changes in the expression of genes involved in the two-component system and cytokinin signaling pathway (*AHK1, AHK3, AHP4* and *ARR1*), homeodomain transcription factors (*WUSCHEL* and *WOX5*), auxin response factor 5 (*ARF5*, Monopteros) and other key regulators of SE *BABY-BOOM* (*BBM*), *LEAFY COTYLEDON1* (*LEC1*), *FUSCA3* (*FUS3*), and *AGAMOUS-LIKE 15* (*AGL15*).

## Results

### SE induction of *C. arabica* var. Typica

**Figure 1:**
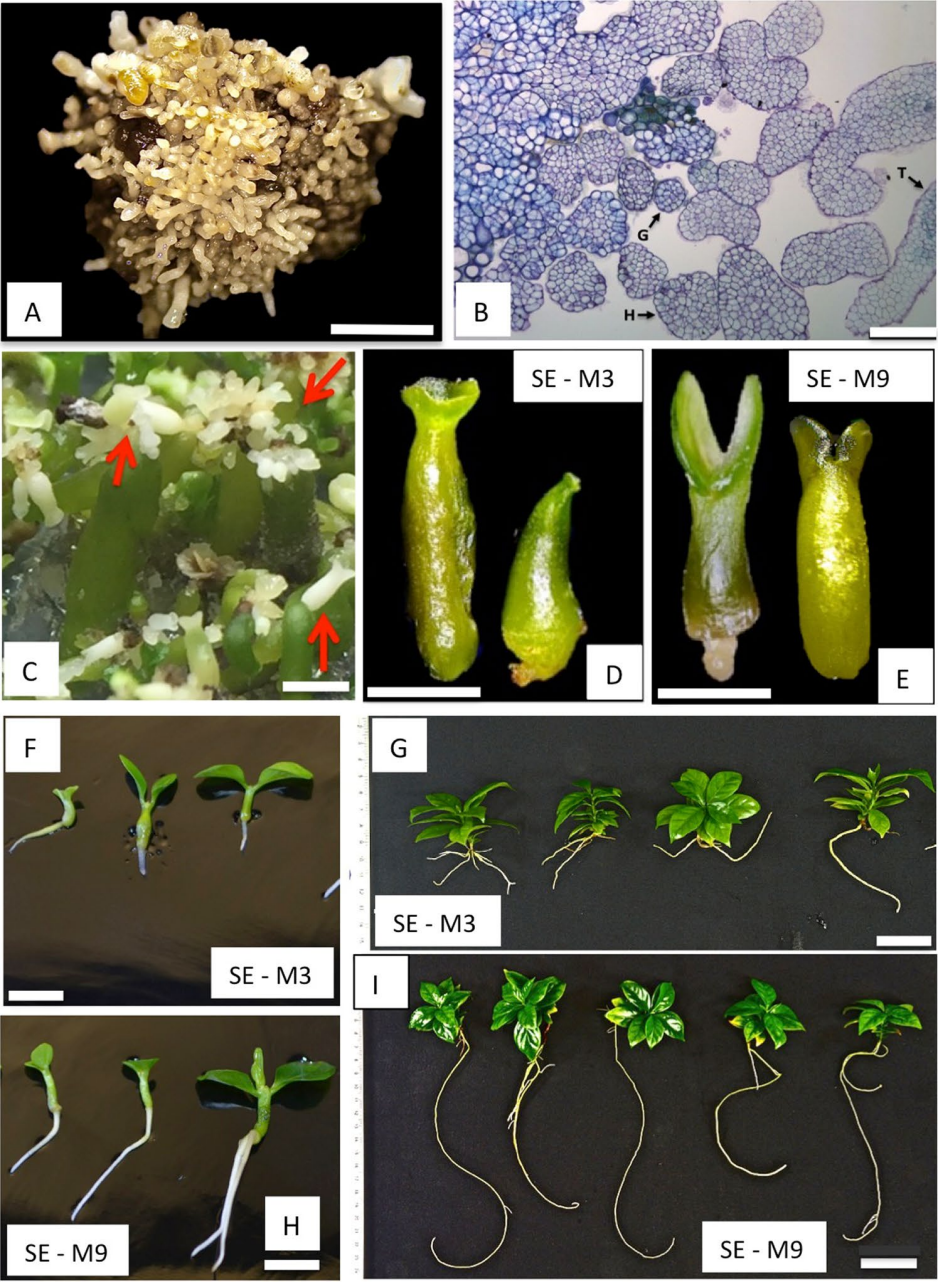
SEs maturation of coffee *C. arabica* var. Typica. (**A**) PEM of *C. arabica* var. Typica at globular and early torpedo stage with high competence of genetic transformation. PEM containing 200 SEs were used to initiate SEs maturation. Bar represents 2 mm. (**B**) Histological analysis of clusters of SEs at globular and early torpedo stage. Bar represents 100 μm. (**C**) First stage of SEs maturation after one month of culture. Secondary SEs were developed at this stage. Bar represents 5 mm. Red arrows indicate secondary SEs. (**D**) SEs at the end of second stage of maturation in SE-M3 non-osmotic medium after one month of culture. Bar represents 2 mm. (**E**) SEs at the end of the second stage of SEs maturation in SE-M9 osmotic medium after one month of culture. Suspensor-like structures at the base of SEs are present. Cotyledons are well developed. Bar represents 1.5 mm. (**F**) Plantlet conversion of SEs developed from SE-M3 non-osmotic medium cultured in SE-G medium after 30, 45 and 60 days from left to right, respectively. Bar represents 5 mm. (**G**) Regenerated plants after 5 months in SE-G, derived from SE-M3 non-osmotic medium. Small root 3.7 cm length and 8 leaves can be appreciated in these plants. Bar represents 3 cm. (**H**) Plantlet conversion of SEs developed from SE-M9 osmotic medium cultured in SE-G medium after 30, 45 and 60 days from left to right, respectively. Bar represents 5 mm. (**I**) Regenerated plants after 5 months in SE-G, derived from SE-M9 osmotic medium. Long pivotal root about 27.6 cm of length and 12 leaves can be appreciated in these plants. Bar represents 3 cm.

Direct SE was induced from leaf explants of *C. arabica* var. Typica with a high potential of propagation, plantlet conversion and genetic transformation. Three months were required to induce the development of SEs to be ready for stable genetic transformation^8^. Briefly, this protocol was followed: leaf explants of coffee cultured in callus induction medium (CIM medium, Van Boxtel et al.^14^) provided proembryogenic masses (PEM) obtained after one month with 93% efficiency of cultivated explants. SEs development was achieved when PEM were subcultured in SE-P medium after two months in culture, with an efficiency ranging from 82 to 100% (Fig. 1A, B).

### SEs maturation is enhanced under osmotic stress

SEs maturation was more efficient in osmotic-stress (SE-M9) (see Material and Methods) in contrast with non-osmotic (SE-M3) (see Material and Methods) solid medium and under light conditions according to the plantlet conversion obtained (Table 1). In the first stage of SEs maturation, secondary SE was induced from the base of initial SEs under osmotic-stress in 83.7% compared to 16.3% of non-stress medium (Fig. 1C). Secondary SEs at early torpedo stage, symmetric and having a suspensor-like structure were separated and individually subcultured to the corresponding fresh medium. 19,000 secondary SEs from osmotic stress (SE-M9 medium) and 6192 SEs derived from non-osmotic medium (SE-M3 medium) were used. Secondary SEs produced in osmotic stress (SE-M9 medium) were characterized by a prominent suspensor-like structure at the early cotyledonary stage, well developed cotyledons, parallels and symmetric, while in non-osmotic medium (SE-M3 medium) secondary SEs produced less prominent suspensor-like structures and altered cotyledon shape (fused-circular) (Fig. 1D, E). At the end of the second stage of maturation, SEs produced in SE-M3 and SE-M9 differed in their phenotype. SEs size at cotyledonary stage was 3.85 ± 0.43 mm in osmotic stress (SE-M9) and 6.0 ± 0.33 mm in non-osmotic stress (SE-M3) (Fig. 1D, E; Table 1). SEs cotyledon size derived from osmotic stress medium (SE-M9) ranged from medium (60%) to small (40%), according to Etienne et al.^1^, contrasting with non-osmotic (SE-M3) that induced large and misshapen (55%) and medium (45%) in size (Table 1). SEs became green in color after three weeks in SE-M9 medium and four weeks in SE-M3 medium. When mature SEs were subcultured individually in SE-G medium, germination was clearly observed after 2 weeks of culture in both treatments. SEs conversion to plantlet was 95.9% achieved in osmotic–stress medium (SE-M9) (18,050 plantlets derived from 19,000 secondary mature SEs) compared to 39.3% in non-osmotic (SE-M3) (2477 plantlets derived from 6192 secondary mature SEs) (Table 1). Differences in root and shoot length were evident after 15, 30 and 45 days respectively of conversion in SE-G medium (Fig. 1F, H).

**Table 1:** SEs maturation efficiency of *C. arabica* var. Typica produced in two different treatments: SE-M3 (non-osmotic) and SE-M9 (osmotic medium).

Evaluated features	SE-M3 Non-osmotic	SE-M9 Osmotic
First stage: Embryogenic clusters (200 SEs at globular stage)	100 (20,000 SEs)	100 (20,000 SEs)
Second stage: Individual SEs at cotyledonary stage derived from secondary SE	6192	19,000
Second stage: Cotyledon shape	Large and misshapen (55%) and medium (45%)	Small (40%) and medium (60%)
Mature SEs showing conversion to plantlets	2477	18,050
Embryo-to-plantlet conversion rate (%)	39.34%	95.9%

### Robustness of plants induced by osmotic stress

Plantlets derived from SEs under osmotic and non-osmotic treatments were subcultured to petri dishes containing SE-G medium for further growth and development. After 5 months in SE-G medium, plantlets were transferred to soil conditions. At this time, the architecture of plants derived from treatments was significantly different. Plants derived from SE-M3 medium generated small, bifurcated roots, 3.7 cm in length and 8 leaves (Fig. 1G). Contrasting with plants derived from SE-M9 medium, that developed long pivotal roots about 27.6 cm length and 12 leaves (Fig. 1I). These plants were successfully established in soil-containing pots and incubated in a growth chamber. Survival rate was 100% in plants derived from osmotic stress medium while 90% was obtained from non-osmotic stress.

**Figure 2:**
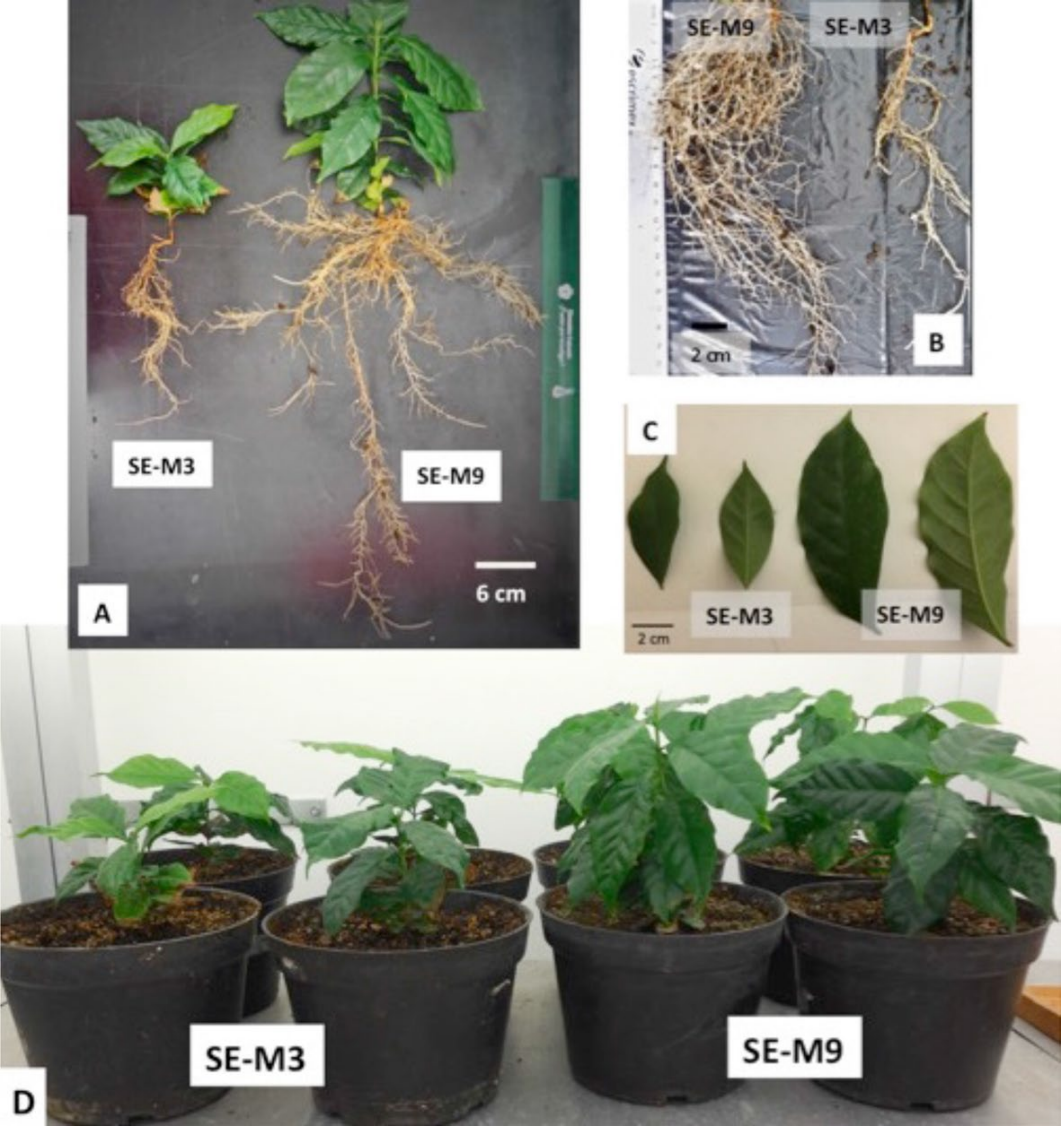
Morphology of plants after eight months in soil conditions under a growth chamber. (**A**) Plants derived from osmotic stress are robustness, with a higher leaf and root area than plants from non-osmotic medium (SE-M3). (**B**) Comparison of root area between SE-M9 and SE-M3 plants. (**C**) Comparison of leaf area between SE-M9 and SE-M3 plants. (**D**) Overview of plants derived from osmotic (SE-M9) and non-osmotic (SE-M3) medium in soil conditions after eight months.

After eight months in soil, as expected from previous morphology, more robust plants developed from SE-M9 osmotic medium. An increase in rooting and leaf area as well as internodes length in plants derived from osmotic stress was observed compared to the non-osmotic treatment. Plants derived from osmotic-stress developed a root area of 105.82 ± 1.06^a^ cm^2^ compared to 11.56 ± 0.08^b^ cm^2^ from non-osmotic stress (Fig. 2A,B). Root length was significantly different: 47.3 ± 0.9^a^ cm were recorded in osmotic stress derived plants while in non-osmotic stress only 23.2 ± 1.24^b^ cm were induced (Table 2). The number of lateral roots from SEs derived from SE-M9 osmotic medium were 18.3 ± 0.13^a^ as compared with 4.7 ± 0.4^b^ in SE-M3 non-osmotic medium. Similarly, lateral root length showed 16.27 ± 9.13^a^ cm in SE-M9 osmotic medium compared with 12.1 ± 4.7^a^ cm from SE-M3 non-osmotic medium (Table 2).

**Table 2:** Morphological differences between plants of *C. arabica* var. Typica regenerated from mature SEs produced in SE-M9 (osmotic medium) and SE-M3 (non-osmotic) medium after eight months.

Evaluated features	Non-osmotic treatment (±SD)	Osmotic treatment (±SD)
Number of leaves	12.0 ± 1.2^b^	22.0 ± 4.0^a^
Length of leaves (cm)	6.1 ± 0.9^b^	13.3 ± 1.33^a^
Leaves width (cm)	2.01 ± 1.7^b^	4.3 ± 0.5^a^
Leaves area (cm^2^)	11.24 ± 0.62^b^	36.31 ± 3.2^a^
Number of ribbing (cm)	10.3 ± 1.7^b^	18.6 ± 1.7^a^
Length ribbing (cm)	1.16 ± 0.32^a^	1.88 ± 0.73^a^
Height (cm)	7.65 ± 1.4^b^	16.7 ± 1.32^a^
Internodes (cm)	8.66 ± 1.15^a^	6.33 ± 1.5^b^
Length of internodes (cm)	0.92 ± 0.11^b^	2.0 ± 0.31^a^
Root length (cm)	23.2 ± 1.24^b^	47.3 ± 0.9^a^
Root area (cm^2^)	11.56 ± 0.08^b^	105.82 ± 1.06^a^
Number lateral root	4.7 ± 0.4^b^	18.3 ± 0.13^a^
Length lateral root (cm)	12.1 ± 4.7^a^	16.27 ± 9.13^a^

Differences in variables among treatments were tested using factorial analysis of variance (ANOVA). Values are means ± standard deviation. In each row, means followed by different letters are significantly different (P < 0.05). SE-M3 non-osmotic medium (control) and SE-M9 osmotic medium. Software ImageJ.

In regard to leaves, plants derived from osmotic stress developed an average of 22.0 ± 4.0^a^ leaves, a leaf area of 36.31 ± 3.2^a^ cm^2^, a length of 13.3 ± 1.33^a^ and a width of 4.3 ± 0.5^a^. In contrast, plants derived from non-osmotic treatment a significant reduction was observed, as they developed only 12.0 ± 1.2^b^ leaves, a leaf area of 11.24 ± 0.62^b^ cm^2^, a length of 6.1 ± 0.9^b^ cm and a width of 2.01 ± 1.7^b^ cm (Table 2) (Fig. 2C). Internodes length were also different, as plants derived from osmotic-stress showed 2.0 ± 0.31^a^ cm in length, while plants derived from non-osmotic treatment showed 0.92 ± 0.11^b^ cm internodal length (Fig. 2D).

### Effect of osmotic stress on SAM and RAM in mature SEs

**Figure 3:**
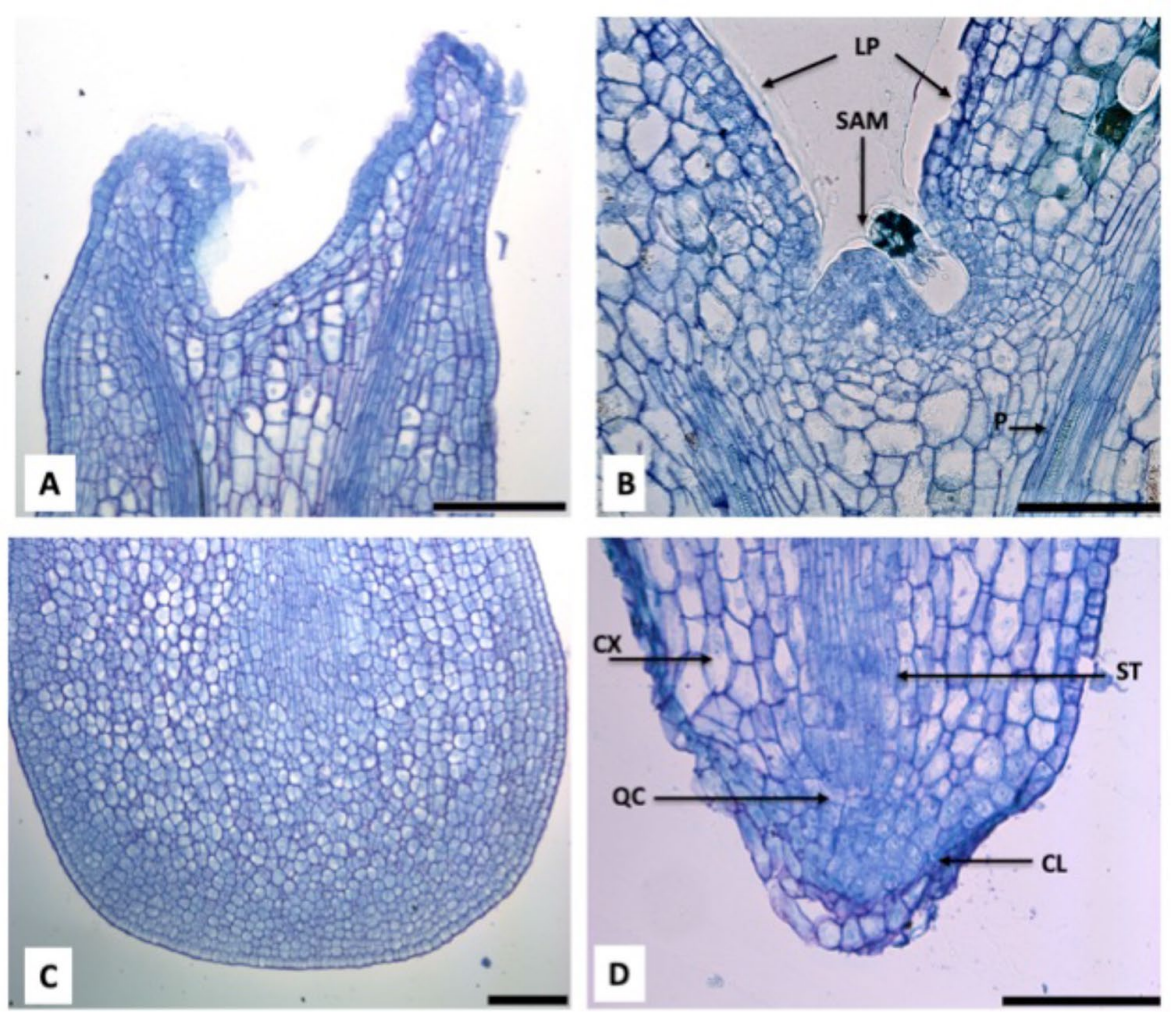
Histological analysis of SEs induced by non-osmotic stress (**A** and **C**) and by osmotic stress (**B** and **D**). (**A**) SEs at cotyledonary stage derived from SE-M3, notice that SAM is not well developed. Bar indicates 100 μm. (**B**) SEs at cotyledonary stage derived from SE-M9 treatment, notice that SAM is well developed. Bar indicates 100 μm. (**C**) SEs at cotyledonary stage derived from SE-M3 treatment, notice that RAM is not developed. Bar indicates 100 μm. (**D**) SEs at cotyledonary stage derived from SE-M9 treatment, notice that RAM is well developed. SAM, shoot apical meristem, LP, leaf primordium. Root apical meristem (RAM). CL, columella, ST, stella, CX, cortex, QC, quiescent center. Bar indicates 100 μm.

SEs at cotyledonary stage grown under osmotic stress and cytokinins developed shoot and root apical meristems in 100% of analyzed samples, while untreated SE did not formed it Fig. 3). Histological analysis of mature SEs induced by osmotic stress revealed that morphology of root apical meristems (RAM) are tiered or closed eudicot, typical of the Rubiaceae family to which *C. arabica* belongs to^19^. Three-tiered differential cells: stella, cortex and columella, were observed in the RAM. Columella cells with 12X18 μm, stella cells 27X5.8 μm, cortex cells 12X18 μm, and quiescent center (QC) 11.76 μm (Fig. 3B, D). On the other hand, embryos derived from non-osmotic medium, apical meristems were absent (Fig. 3A, C).

### Differentially expressed key factors involved in SEs maturation of *C. arabica* var. Typica under osmotic stress

**Figure 4:**
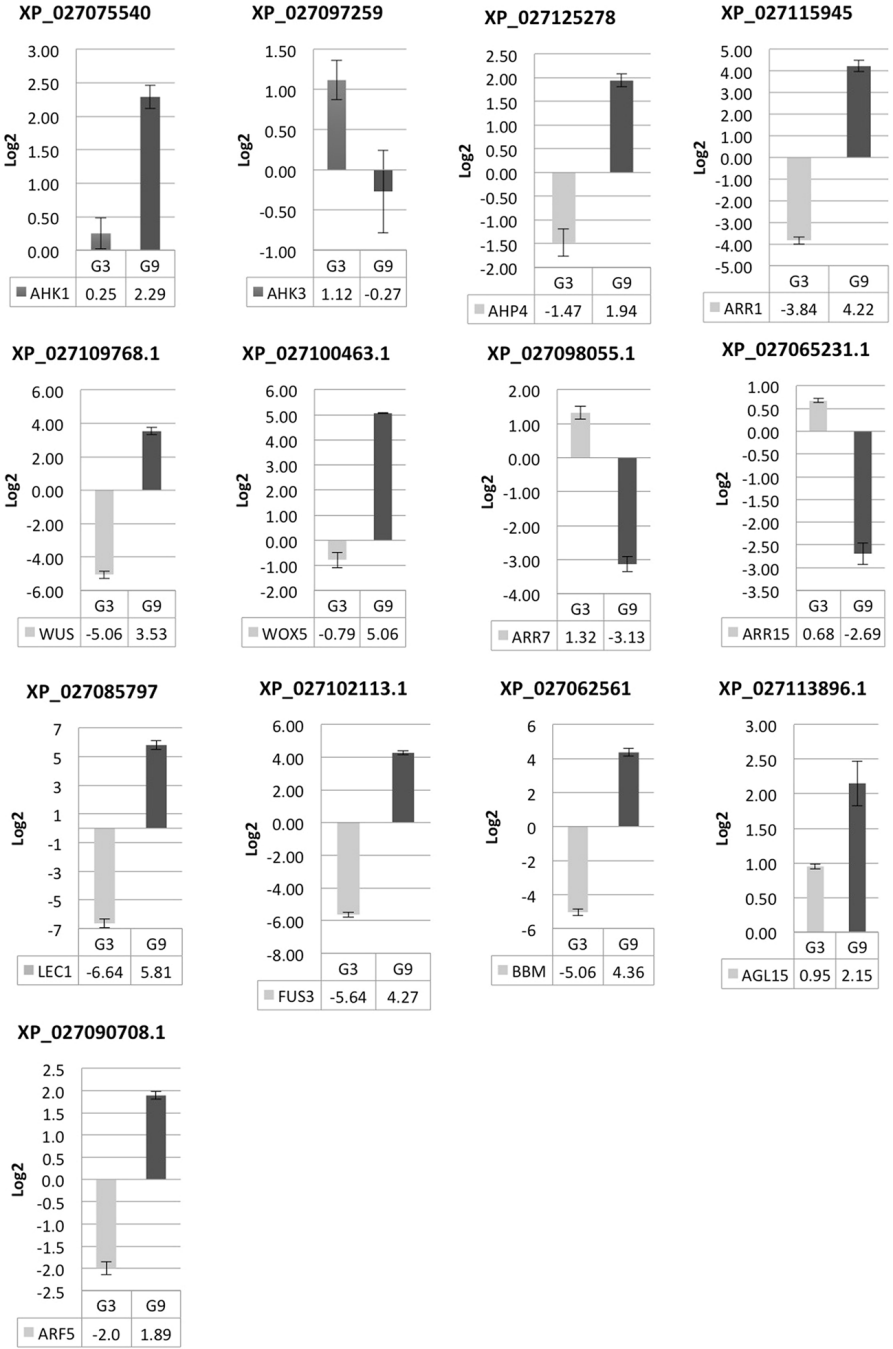
Gene transcript relative expression (Log2) during SEs maturation in *C. arabica*. Relative expression of genes*, AHK1, AHK3, AHP4, ARR1, ARR7, ARR15, WUSCHEL, WOX5, ARF5, LEC1, FUS3, BBM* and *AGL15.* Genes were normalized with, *Actine, RP29* and *24S*. Each qPCR was conducted with three independent biological replicates. M3, SE-M3; MS medium supplemented with 0.2 mg/L BAP, 0.1 mg/L kinetin, 1% glucose, 3.0 g/L gelrite (− 0.49 MPa), pH 5.8, M9, SE-M9; MS medium supplemented with 0.2 mg/L BAP, 0.1 mg/L Kinetin, 1% glucose, 9.0 g/L gelrite (− 1.47 MPa).

**Figure 5:**
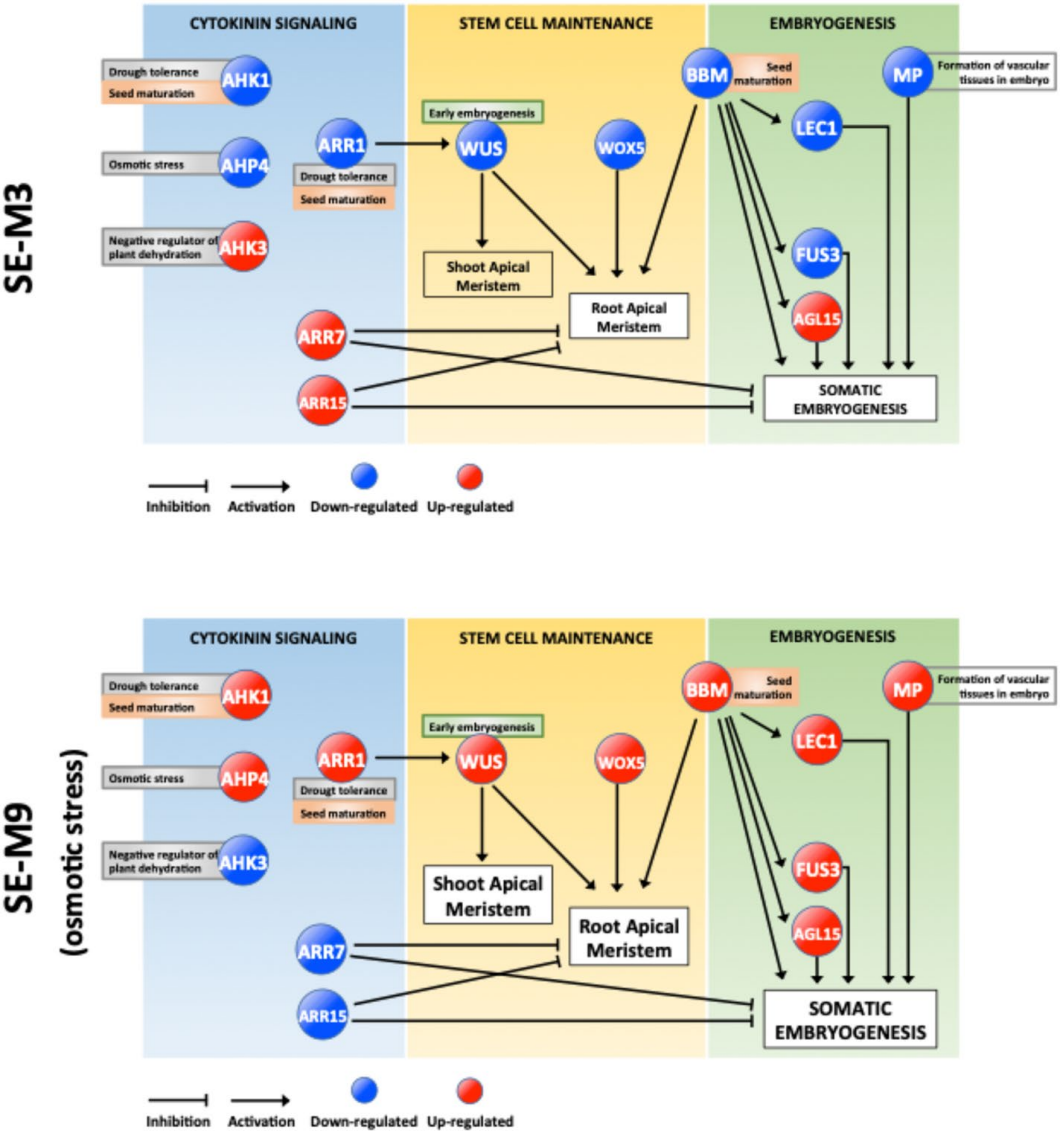
A model of SEs maturation regulatory network in two different media. Differences in shoot and root apical meristems observed in the SEs are related to expression changes of key genes involved in cytokinin signaling, stem cell maintenance and embryogenesis. Initial samples were SEs from PEM at globular and early torpedo steges. The second sample were secondary SEs at cotyedonary stage in the second month of maturation. SEs were cleary developed with specific shape in osmotic stress medium contrasting with non-osmotis stress medium.

To understand the molecular mechanism allowing SAM and RAM formation under osmotic stress a STRING-based bioinformatic analysis with high confidence (0.700) based on *C. arabica* homologous sequences in the *Arabidopsis thaliana* genome were performed (Supplementary Fig. 2). A set of two component system (*AHK1*), cytokinin signaling (*AHK3, AHP4, ARR1*) and auxin signaling (*ARF5*) related coding genes were evaluated, and according with the theoretical gene network in *Arabidopsis*, those proteins interact with *WUSCHEL* and *WOX5* homeodomains and morphogenic genes, such as *BBM, LEC1, FUS3* and *AGL15*, involved in meristem maintenance and embryogenesis (Figs. 4, 5, Fig. S2). As expected, expression levels of *AHK1* (*histidine kinase* 1)*,* that functions as an osmosensor and detects water stress was upregulated in SE-M9 medium (osmotic stress) 2.29 times compared to 0.25 in SE-M3 medium. *AHK3* (*histidine kinase 3*) was downregulated in SE-M9 medium (− 0.27) as compared to in SE-M3 medium (1.11). Levels of expression of the *histidine-containing phosphotransfer*, *AHP4*, was upregulated (1.94) in SE-M9 medium and downregulated (− 1.47) under normal SE-M3 medium conditions. *ARR7* and *ARR15*, two component response regulators type-A, were downregulated in SE-M9 medium, (− 3.13 and − 2.69, respectively) and upregulated (1.32 and 0.69, respectively) in SE-M3 medium. A response regulator type B, ARR1, was upregulated (4.22) in SE-M9 and downregulated (− 3.83) in SE-M3 medium. *WUSCHEL*, a homedomain-like superfamily protein was upregulated (3.53) in SE-M9 and downregulated (− 5.05) in SE-M3 medium. *WOX5*, a *WUSCHEL* related homeobox, was upregulated (5.06) in SE-M9 medium and downregulated (− 0.78) in SE-M3 medium. ARF5, an auxin response factor, was upregulated (1.89) in SE-M9 medium and downregulated (− 2.0) in SE-M3 medium.

Additionally, *BBM*, an AP2-like ethylene-responsive transcription factor, was upregulated (4.36) in SE-M9 medium and downregulated (− 5.6) in SE-M3 medium. *LEC1,* a nuclear transcription factor Y subunit B-9, was upregulated (5.81) in SE-M9 medium and down regulated (− 6.64) in SE-M3 medium. *FUS3*, a regulator of gene expression during late embryogenesis, was upregulated (4.27) in SE-M9 medium and downregulated (− 5.64) in SE-M3 medium. *AGL15*, a member of MADS domain family of regulatory factors, was upregulated (2.15) in SE-M9 medium and upregulated as well (0.94) in SE-M3 medium (Figs. 4, 5, and Table S2).

According to the gene expression analysis of somatic embryo maturation of *C. arabica*, a molecular model is presented, which indicates how the upregulation of type A response regulators (*ARR7-ARR15*) and *histidine-kinase 3* (*AHK3*) under non-osmotic treatment inhibits SAM and RAM development and consequently somatic embryo maturation. In this treatment, genes involved in drought tolerance and seed maturation *AHK1, AHP4* and *ARR1* were repressed, blocking the activation of genes necessary for stem cell maintenance (*WUS-WOX5*) and master regulators of embryogenesis (*LEC1-FUS3-BBM-AGL15*) (Fig. 5). The opposite effect under osmotic stress treatment (SE-M9), where *AHK1, AHP4* and *ARR1* were upregulated allowing activation of key SE genes such as *BBM, LEC1, MP,* and *FUS3* leading to acquire embryogenic competence, and robustness in regenerated plants (Fig. 5). Further analysis of each component is required to clearly elucidate this developmental phenomenon.

## Discussion

SE induced from leaf explants has been the most widely used target tissue in coffee micropropagation and stable genetic transformation^1^. Published protocols have shown that SE induction and proliferation is time consuming, ranging from 9 to 20 months until they can be used for stable genetic transformation^8,20,21^. In most of these reports, SE regeneration to plants in *C. arabica* has been shown to be more difficult and time consuming than in *C. canephora* P*.* As noted by several authors, auxins negatively affect SE development in coffee^14–16^. Our strategy was to simulate natural processes occurring during embryo development in coffee seeds. We hypothesized that osmotic stress and the presence of cytokinins, are both necessary for a correct SEs development. This is supported by cytokinin signaling, as it has shown that plays a critical role during root and stem cell niche formation allowing RAM system initiation in SE^17,18^.

Our qRT-PCR analyses revealed differential expression of key factors allowing the enhanced plant regeneration in SE-M9 treatment in comparison to the SE-M3 (Fig. 4, Table S1). According to the gene network devised in STRING with a high confidence threshold (0.700) based on *C. arabica* homologous genes present in *Arabidopsis* (Fig. S2) and transcriptional gene expression analysis by qPCR, SE maturation is related to 3 main processes: stem cell maintenance, cytokinin signaling, and embryogenesis (Fig. 5). Under these conditions, the coding genes involved in the two-component signaling system and cytokinin signaling, such as histidine-kinase1 (*AHK1*), it was upregulated in SE-M9 treatment. *AHK1* has functions as osmosensor, improvement for drought tolerance in *Arabidopsis*^22–24^ and plays a unique role in the regulation of desiccation processes during seed maturation^25^. *AHK1* feeds phosphates to the phosphorelay-integrated histidine phosphotransfer protein *AHP4*, that in our analysis it was upregulated in SE-M9 treatment. Singh et al.^26^, have shown upregulation of *AHP4* under osmotic stress treatment, whereas other members of this family (*AHP1, AHP2* and *AHP3*) were downregulated. In the two-component signaling system, the final transcriptional response occurs via transcription factors. Two type-A response regulators, *ARR7* and *ARR15*, were downregulated in SE-M9 treatment. *ARR7* and *ARR15* negatively regulates cytokinin signaling and suppress the size of the meristem^27,28^. Overexpression of *ARR7* or *ARR15* inhibit RAM formation and SEs regeneration, as well as in *ahk2/ahk4* or *ahk3/ahk4* mutants, in which cytokinin signaling was abolished^17^. In our study, a B-type response regulator, *ARR1*, was upregulated in SE-M9. *ARR1* works as a transcriptional activator of *WUSCHEL*^29^, DNA repair, ROS scavenging, oxidative stress, and drought tolerance^30–32^.

*WUSCHEL* a homedomain-like superfamily protein was upregulated with the SE-M9 treatment and downregulated in SE-M3 medium. Cytokinin signaling and B-type responsive factors activate *WUSCHEL* expression^29,33–35^. *WUSCHEL* plays a central role during early embryogenesis and requires to specify stem cell fate in meristems, such as shoot apical meristem (SAM). *WUSCHEL* has been essential for RAM and SAM initiation and embryonic shoot–root axis establishment^17,36^.

Similarly, *WOX5*, a *WUSCHEL*-related homeobox protein, was upregulated in SE-M9 treatment and downregulated under normal conditions (Figs. 4, 5). *WOX5* is a transcription factor which may be involved in the differentiation and maintenance of the stem cells in the RAM^17,37,38^.

The auxin responsive factor, *ARF5*, was upregulated in SE-M9 (osmotic stress) treatment and downregulated in normal conditions (Fig. 4). *ARF5* mediates embryo axis formation and vascular tissue differentiation^39–41^. *Arabidopsis* mutants in *arf1*, *arf5* and *arf7* displayed a significant reduction in capacity and efficiency of SEs maturation^42^. Otherwise, overexpression of *ARF5* results in null SEs development^42^. García-Gómez et al.^43^, proposed a dynamic regulatory network model to understand the interaction between auxin and cytokinin signaling pathways and some transcriptional regulators important in the root apical meristem (RAM) of *A. thaliana*. Among genes that were analyzed, *ARF5* was showed to be essential for *WOX5* activity.

The master regulator of SE, *BBM*, is a transcription factor of the AP2/ERF family, which was upregulated in SE-M9 (Figs. 4, 5). *BBM* promotes cell proliferation, differentiation and morphogenesis, specifically during embryogenesis. BBM-induced embryogenesis relies on transcriptional activation of *LEC1, LEC2, FUS3* and *AGL15* genes^44^. Members the AP2 gene family (cytokinin response factors CRFs), closely related to *BBM,* are transcriptionally upregulated by cytokinin signaling^45^. CRFs mutant combinations have demonstrated to be lethal to embryos^46^. *LEC1*, a nuclear transcription factor Y subunit B-9, was upregulated in SE-M9 (Figs. 4, 5). *LEC1* is a transcriptional activator required for both embryo maturation and cellular differentiation. *FUS3*, a regulator of gene expression during late embryogenesis, was upregulated in SE-M9 (Figs. 4, 5, and Table S2). *FUS3* is a regulator of gene expression during late embryogenesis. *Arabidopsis* mutants in *lec1, lec2, fus3* show total SE repression due to the loss of desiccation tolerance^47^.

*AGL15*, a member of MADS-box domain family of regulatory factors was upregulated in SE-M9 treatment, and also upregulated as well in normal conditions (Figs. 4, 5, Table S2). *AGL15* is preferentially expressed during embryogenesis and gibberellic acid catabolism.

The above-mentioned differential expression of key genes is similar to that detected during the successive stages of seed maturation in *C. arabica*. Dussert et al*.*^48^, reported that one coding gene for *AHK3* was upregulated in subsequent stages of seed maturation of *C. arabica*. In this transcriptional analysis it was found other upregulated genes related with cytokinin signaling: one gene for *AHK4*, three for *AHP1*, four type-B response regulators and five type-A response regulators. In the case of transcription factors, *BBM2* and *ARR2*, a redundant gene of *ARR1*, were differentially upregulated in the last four stages of maturation, a similar behavior was observed with *FUS3* which was differentially upregulated in two stages of maturation (4 and 5 out of 7)^48^. This genetic network represents a preliminary evidence in the understanding of SEs maturation process of *C. arabica* var. Typica. It is conceivable that epigenetic reprogramming could have also occurred during the maturation developmental process of SEs. Changes or modifications in DNA methylation patterns are linked with regulatory mechanisms of master regulators genes of SE such as *LEC1, LEC2, FUS3* and *AGL15*^49^. Further analysis of each component is required to clearly elucidate this developmental phenomenon.

In this work we were able to reach up to 95.9% of germination and complete plant regeneration with SEs under osmotic stress treatment in contrast with 39.34% in untreated SEs. The effect of different water potentials in SE conversion in *C. arabica* was reported. Etienne et al.^3^ analyzed the developmental process of zygotic and SEs of coffee *C. arabica* grown in temporary immersion bioreactors, a system with changing water potential depending on immersion cycles. They found that a higher conversion rate of germination of zygotic embryos (96%) versus SEs (55%). Conversion of SE was increase up to 91% using a bigger bioreactor with differences in water characteristics (water potential). It is important to notice that shoot and root apical meristems were developed compared to untreated SE. Failure in forming proper SAM and RAM in SEs was published elsewhere^1,3,7,9,13,50^. The importance of the apical meristems in plant growth and development has long been recognized^51^, but in woody plants, little is known about meristems formation during SE^11^. Most of the studies on the molecular network regulating embryo development have been made in *Arabidopsis*^52^.

In *C. arabica,* a mutation called *laurin*a occurred in highland Ethiopia, alters shoot apical meristem (SAM) dimensions, resulting in smaller leaf primordia and incipient internodes^53^. In our work, under osmotic stress and cytokinin presence we showed the activation of SAM and RAM dimensions and SEs conversion to plantlet (Fig. 3B,D).

If whether or not our results are related with *laurina* mutation, analogies with the observations reported in *Arabidopsis* in early timing within plastochron reducing SAM dimensions for some genes such as *AXR1, ARGOS, ANT*, related to auxin regulation pathway may explain the negative role in SEs maturation of auxins^54–56^.

Regenerated plants derived from osmotic stress showed an increased fitness including vigorous roots, increased leaf area, and stem length, leading to an increased anchorage, nutrition and productivity. In economic terms, a reduction of costs (up to 80%) in plant production derived from somatic embryos subjected with osmotic stress with improved agronomic performance is expected**.**

Additionally, development of SAM and RAM in mature SEs derived from osmotic treatment can be considered a morphological marker for quality control in SEs development in *C. arabica* var. Typica.

## Conclusions

Desiccation using high content of gelrite in the presence of cytokinins in SEs plays an important role for maturation and conversion to plantlet of *C. arabica* var. Typica.

Reprogramming of genes involved in shoot and root apical meristems occurred during maturation of SEs under osmotic stress, enhancing robustness of converted plants.

Development of SAM and RAM in mature SEs derived from osmotic treatment can be considered a morphological marker for quality control in SEs development in *C. arabica* var. Typica.

Bioinformatic tools such as STRING achieved improvement in the understanding of molecular mechanisms involved in SEs maturation.

The application of strategies derived from this knowledge can be applied in tissue culture media composition and morphological and physiological changes in the expected phenotype.

## Methods

### Induction of SE in *C. arabica* var. Typica

SE in *C. arabica* var. Typica (INIFAP-Tapachula, Chiapas), was induced from leaf explants, derived from eight-months old trees. Explants were disinfected by gasification in a vacuum chamber with a mixture of 50 mL of sodium hypochlorite from a commercial bleach (1.2% active chlorine) and 50 mL HCl 6 N for 15 min and washed four times with sterile distilled water^57^. Disinfected leaves were cut into 1 cm^2^ pieces and cultured on the callus induction CIM medium as described by Van Boxtel et al.^14^: half-strength Murashige and Skoog medium^58^, 30 g/L sucrose, 100 mg/L casein hydrolysate, 400 mg/L malt extract, 10 mg/L thiamine, 1 mg/L nicotinic acid, 1 mg/L pyridoxine, 1 mg/L glycine, 100 mg/L myo-inositol, 0.5 mg/L 2.4-D, 1 mg/L indole-3-butyric acid (IBA), 2 mg/L 2-isopentenyladenine (2-iP), and solidified with 2.4 g/L gelrite, pH was adjusted to 5.8 before autoclaving. After two months in dark, PEM were sub-cultured into SE-P medium, as described Van Boxtel et al.^14^, for SE propagation with slight modifications, half-strength MS salts medium^58^, 30 g/L sucrose, 200 mg/L casein hydrolysate, 800 mg/L malt extract, 60 mg/L adenine-free base, 1 mg/L 2.4-D and 4 mg/L BAP, pH 8.0 and solidified with 3.2 g/L gelrite.

### SEs maturation of *C. arabica* var. Typica

To evaluate the role of two component system and cytokinin signaling coupled with osmotic stress in SEs maturation of coffee *C. arabica,* two treatments were evaluated: a) SE-M3: MS medium supplemented with 0.2 mg/L BAP, 0.1 mg/L Kinetin, 1% glucose, 3.0 g/L gelrite (− 0.49 MPa), pH 5.8; and b) SE-M9: MS medium supplemented with 0.2 mg/L BAP, 0.1 mg/L Kinetin, 1% glucose, 9.0 g/L gelrite (− 1.47 MPa), pH 5.8.

The protocol of SEs maturation included two stages: In the first one, PEM (with soft consistency, 0.7 to 10 mm^2^ size), derived from CIM medium, were used as source of SEs. 100 PEM (each one containing about 200 SEs at globular and torpedo stage) yielding a total of 20,000 SEs. PEM were individually transferred to respective cultured medium (SE-M3 and SE-M9), using forceps and slightly smash to allow SEs to get contact with the medium. 10 PEM were cultured in each plastic petri dish (90X20 mm, Phoenix biomedical product). PEM were incubated at 25 ± 2 °C, under a 12/12 h photoperiod at 50 µmol/m^2^-s irradiance provided by fluorescent lamps T8 Phillips P32T8/TL850 combined with natural sunlight, producing a unique light spectrum (Figure S1). In the second stage, we selected and subcultured only SEs derived from secondary SE developed from SEs from the first stage. SEs at early torpedo stage, symmetric and having a suspensor-like structure were separated, and individually subcultured to the corresponding fresh medium (SE-M3 or SE-M9), 25 SEs in each plate, for one month until cotyledonary stage developed according to Valencia-Lozano et al*.*^8^.

### Conversion of SEs to plantlets of *C. arabica* var. Typica

Secondary SEs subjected to osmotic stress (SE-M9) (19,000 total) and non-osmotic stress (SE-M3) (6192 total) in cotyledonary stage, green color, symmetric and having a suspensor-like structure were subcultured to plantlet conversion in SE-G medium: MS medium^58^ 30 g/L sucrose, 1 g/L activated charcoal, 3 g/L gelrite and smoke water 0.5% (karrikins)^59^. Incubation was under the same conditions mentioned in SEs maturation. Regenerated plantlets were subcultured in flasks with SE-G medium until plants reached 10 leaves and 27 cm root length. Regenerated plants were potted in vermiculite and soil (1:1 v/v), incubated in a plant growth chamber at 50 µmol/m^2^-s irradiance provided by T8 Phillips P32T8/TL850 fluorescent lamps with a photoperiod of 16/8 h and temperature of 19 to 23ºC, as described by Valencia-Lozano et al.^8^. Efficiency of SEs maturation was calculated according to the number of SEs converted to plantlets derived from SEs at cotyledonary stage produced at the end of the second stage of maturation.

### Histological analysis of SEs

Randomly chosen SEs at different stages (5 per treatment) were collected and fixed in FAE (5% formaldehyde, 10% acetic acid, 50% ethanol), followed by dehydration in a series of ethanol dilutions (20%, 40%, 60%, 80% and 100% ethanol) for 2 h each. Samples were embedded in Technovit 7100 (Heraeus Kulzer) according to the manufacturer instructions. Sections (14 μm) were obtained on a rotary microtome (Reichert-Jung 2040; Leica). Tissue sections were stained with a 0.02% Toluidine Blue solution (HYCEL, Zapopan, Mexico), samples were stained for 3 min, washed with distilled water for 1 min, and air dried. Pictures were taken using a DM6000B microscope (Leica).

### Isolation of RNA and gene expression analysis

Total RNA from SEs cultured in osmotic-medium (SE-M9) and non-osmotic medium (SE-M3) was isolated using Trizol (Invitrogen, Carlsbad, CA, USA). RNA concentration was measured by its absorbance at 260 nm, ratio 260 nm/280 nm was assessed, and its integrity confirmed by electrophoresis in agarose 2% (w/v) gels. Samples of cDNA were amplified by PCR using SYBR Green qPCR (BioRad) in Real-Time PCR Systems (CFX96 BioRad). The reference genes in this work were *ACT, 24S* and *RPL39*, according to Freitas et al. (2017) applied for qPCR analysis of embryogenic calli, somatic embryos and callus of coffee. Retfinder, NormFinder, Bestkeeper and Delta-Ct were used in this analysis. Three replicates were done with these reference genes, calculated the relative expression, and weighted ct, and next a delta ct in each gene analyzed and relative amount of target gene expression using the 2^−ΔΔCT^ method^60^. qPCR analysis was based on at least three biological replicates for each sample with three technical replicates and control treatment SE-P. To select genes involved in SE development process, a gene network with a high confidence (0.700) was performed with STRING (v11.0, http://string-db.org), based on *C. arabica* homologous genes present in *A. thaliana* genome. The selected genes were: Two component system and cytokinin signaling genes, *AHK1, AHK3, AHP4, ARR1, ARR7, ARR15,* auxin signaling; *ARF5* (monopteros), homedomains; *WUSCHEL, WOX5*; master regulator of embryogenesis, *LEC1, BBM, FUS3*, and *AGL15* (Table S1, Figure S2). Gene identifier was made according to UNIPROT (http://www.uniprot.org), NCBI (http://www.ncbi.nlm.nih.gov) database. Sequences of all genes were analyzed from *A. thaliana* using blastN, blastP in the coffee genome homologous sequences. Homologous sequences in *C. arabica* genome greater than 40% in protein sequence with *A. thaliana* were considered. Proteins of *C. arabica* homologous with *A. thaliana* aligned by BlastP were identified as: (XP_027075540, XP_027097259, XP_027125278, XP_027115945, XP_027098055.1, XP_027065231.1, XP_027100463.1, XP_027109768.1, XP_027090708.1, XP_027085797, XP_027062561, XP_027102113.1, XP_027113896.1) (Table S1). Oligonucleotides were designed to qPCR (2^−ΔΔCT^ method analysis) gene expression or transcriptional analysis (Table S1).

## Supplementary Information

Supplementary Information 1.

Supplementary Information 2.

Supplementary Information 3.

Supplementary Information 4.

## Abbreviations

SE: Somatic embryogenesis
SEs: Somatic embryos
SAM: Shoot apical meristem
RAM: Root apical meristem
BAP: 6-Benzylaminopurine
2,4-D: 2,4-Dichlorophenoxyacetic acid
