# Supplementary material for: Osmotic stress-induced somatic embryo maturation of coffee Coffea arabica L., shoot and root apical meristems development and robustness
Source: Sci Rep. 2021 May 6;11:9661. doi: 10.1038/s41598-021-88834-z (PMC8102543; doi:10.1038/s41598-021-88834-z)
Supplement: Supplementary file 1 — Supplementary Information 1. [file 41598_2021_88834_MOESM1_ESM.pdf]

Supplementary Table 1. Sequence primer qPCR and protein function of genes analyzed during SEs in *C. arabica* var. Typica.

**Gene**	**ID AT (*A. thaliana*)**	**ID XP (*C. arabica*)**	**Protein function**	**% ID At/Ca**	Primer
AHK1	AT2G17820.1	XP_027075540	Histidine kinase 1; Functions as an osmosensor histidine kinase that detects water stress and transmits the stress signal to a downstream MAPK cascade.	64.07	5´ CTTAGCAGCGGGAAGTGTTC 3´<br>3´ CCATGCAAGCGAGTAGTTGA 5´
AHK3	AT1G27320.1	XP_027097259	Histidine kinase 3; Cytokinins (CK) receptor related to bacterial two-component regulators.	67.12	5´ TGGAAGATGGAGAGGATTGG 3´<br>3´ ATTCCCACGATCTTCCTGTG 5´
AHP4	AT3G16360	XP_027125278	*Arabidopsis thaliana* histidine phosphotransfer proteins (AHPs). Encodes AHP4, a histidine-containing phospho-transmitter involved in Histidine (His)-to-Aspartate (Asp) phosphorelay signal transduction. AHP4 is one of the six	70.34	5´ TCAACTGCGACGAAAACTTG 3´<br>3´ GGTTCTTCTCCAGTGCTTGC 5´
ARR1	AT3G16857.2	XP_027115945	Two-component response regulator ARR1; Transcriptional activator that response regulator involved in His-to-Asp phosphorelay signal transduction system.	52.11	5´ TTGCCCGGTAATAGTTTTGC 3´<br>3´ CATTTTGCATACCCCAATCC 5´
ARR7	AT1G19050.1	XP_027098055.1	Arabidopsis response regulator (ARR) family, most closely related to ARR15. A two-component response regulator protein containing a phosphate accepting domain in the receiver domain but lacking a DNA binding domain in the output domain.	63.35	5´AGGGTTTGGCAAGAGAAAATTG 3´<br>3´AGTTTGTGTTGGTGAGGAG 5´
ARR15	AT1G74890.1	XP_027065231.1	Two-component response regulator ARR15; Functions as response regulator involved in His-to-Asp phosphorelay signal transduction system. Phosphorylation of the Asp residue in the receiver domain activates the ability of the protein to promote the transcription of target genes.	50.85	5´ GTGGGTTGTGGATTTTGGTG 3´<br>3´GTATGAGCTGAGGTGAGATGAG 5´
WUS	AT2G17950.1	XP_027100463.1	Homeodomain-like superfamily protein; Transcription factor that plays a central role during early embryogenesis, oogenesis and flowering. Required to specify stem cell identity in meristems, such as shoot apical meristem (SAM).	40.4	5´ CCGGTCCCAGAATTAGAAACTC 3´<br>3´GAATAACCCACGTTGCCATTG 5´
WOX5	AT3G11260.1	XP_027109768.1	WUSCHEL related homeobox 5; Transcription factor, which may be involved in the specification and maintenance of the stem cells (QC cells) in the root apical meristem (RAM)	58.54	5´ TCGCAGAGTTTCGATTGATG 3´<br>3´ GCTGCTTCTCTGCCTCTGAT 5´
ARF5	AT1G19850.1	XP_027090708.1	Transcriptional factor B3 family protein / auxin-responsive factor AUX/IAA-related; Auxin response factors (ARFs) are transcriptional factors that bind specifically to the DNA sequence.	58.01	5´CAATTGAGCGGATGTTTGGAC 3´<br>3´TCCACATACACCAGTTTCCAG 5´
LEC1	AT1G21970.1	XP_027085797	Nuclear transcription factor Y subunit B-9; Transcriptional activator of genes required for both embryo maturation and cellular differentiation. Sequence is similar to HAP3.	76.47	5' CCAGGAATGTGTATCGGAGTAC 3´<br>3´GAAAGCGGTGGAGATATAGGG 5´
BBM	AT5G17430	XP_027062561	AP2-like ethylene-responsive transcription factor BBM; Transcription factor that promotes cell proliferation, differentiation and morphogenesis, especially during embryogenesis	41.53	5´ TTCAACCCCAACGAGATCAG 3´<br>3´GTTGTAGTTCTCCTTCCAGTCC 5´
FUS3	AT3g26790.1	XP_027102113.1	Transcriptional factor with high similarity to the B3 region of the VP1/ABI3-like proteins. Present in many seed-specific promoters.	44.92	5´GGCTTACGACATGGAGACTAC 3´<br>3´GCATTTATCTCCGACTCAGGG 5´
AGL15	AT5G13790.1	XP_027113896.1	AGL15 (AGAMOUS-Like 15) is a member of the MADS domain family of regulatory factors. Is preferentially expressed during embryogenesis, may play a role during post-germinative development.	55.77	5´ TGGGAGGAAGAATTCTGGTG 3´<br>3´CTAACAGGCAAACACGCTGA 5´
