# Supplementary material for: Osmotic stress-induced somatic embryo maturation of coffee Coffea arabica L., shoot and root apical meristems development and robustness
Source: Sci Rep. 2021 May 6;11:9661. doi: 10.1038/s41598-021-88834-z (PMC8102543; doi:10.1038/s41598-021-88834-z)
Supplement: Supplementary file 2 — Supplementary Information 2. [file 41598_2021_88834_MOESM2_ESM.doc]

**Supplementary Table 2. Genes involved in SEs maturation of *C. arabica* identified from differentially expressed genes (DEGs).**

| **Non-Smotic stress** | **Smotic stress** |  | **GENE ID** | **Annotation in the** |
| --- | --- | --- | --- | --- |
| **M3** | **M9** |  |  | ***Coffea arabica* genome** |
| 0.25 | 2.29 |  | AHK1 | XP_027075540 |
| 1.12 | -0.27 |  | AHK3 | XP_027097259 |
| -1.47 | 1.94 |  | AHP4 | XP_027125278 |
| -3.84 | 4.22 |  | ARR1 | XP_027115945 |
| 1.32 | -3.13 |  | ARR7 | XP_027098055.1 |
| 0.68 | -2.69 |  | ARR15 | XP_027065231.1 |
|  |  |  |  |  |
| -2 | 1.89 |  | ARF5 | XP_027090708.1 |
|  |  |  |  |  |
| -5.06 | 3.53 |  | WUS | XP_027100463.1 |
| -0.79 | 5.06 |  | WOX5 | XP_027109768.1 |
| -6.64 | 5.81 |  | LEC1 | XP_027085797 |
| -5.64 | 4.27 |  | FUS3 | XP_027102113.1 |
| -5.06 | 4.36 |  | BBM | XP_027062561 |
| 0.95 | 2.15 |  | AGL15 | XP_027113896.1 |


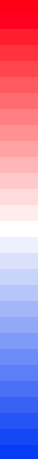


-7 0 7

*SE-M3: Embryogenic medium with 3 g/L gelrite; SE.M9: Embryogenic medium with 9 g/L gelrite. See text and Table S1 for the identification of genes.
