# Supplementary material for: Osmotic stress-induced somatic embryo maturation of coffee Coffea arabica L., shoot and root apical meristems development and robustness
Source: Sci Rep. 2021 May 6;11:9661. doi: 10.1038/s41598-021-88834-z (PMC8102543; doi:10.1038/s41598-021-88834-z)
Supplement: Supplementary file 3 — Supplementary Information 3. [file 41598_2021_88834_MOESM3_ESM.pdf]

Osmotic stress-induced somatic embryo maturation of coffee Coffea arabica L, shoot and root apical meristems development and robustness.

Eliana Valencia-Lozano[1], Jorge E. Ibarra[1], Humberto Herrera-Ubaldo[3], Stefan De Folter[3], José L. Cabrera-Ponce[2*]

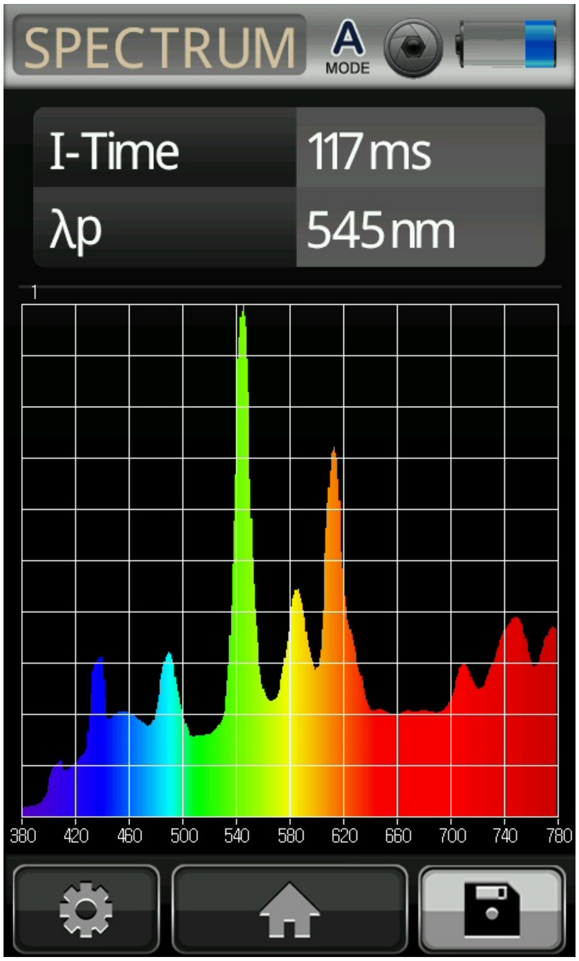

Supplementary Figure 1. Light spectrum used for SEs maturation and conversión to plantlet of C. arabica var. Typica.

Osmotic stress-induced somatic embryo maturation of coffee Coffea arabica L, shoot and root apical meristems development and robustness.

Eliana Valencia-Lozano[1], Jorge E. Ibarra[1], Humberto Herrera-Ubaldo[3], Stefan De Folter[3], José L. Cabrera-Ponce[2*]

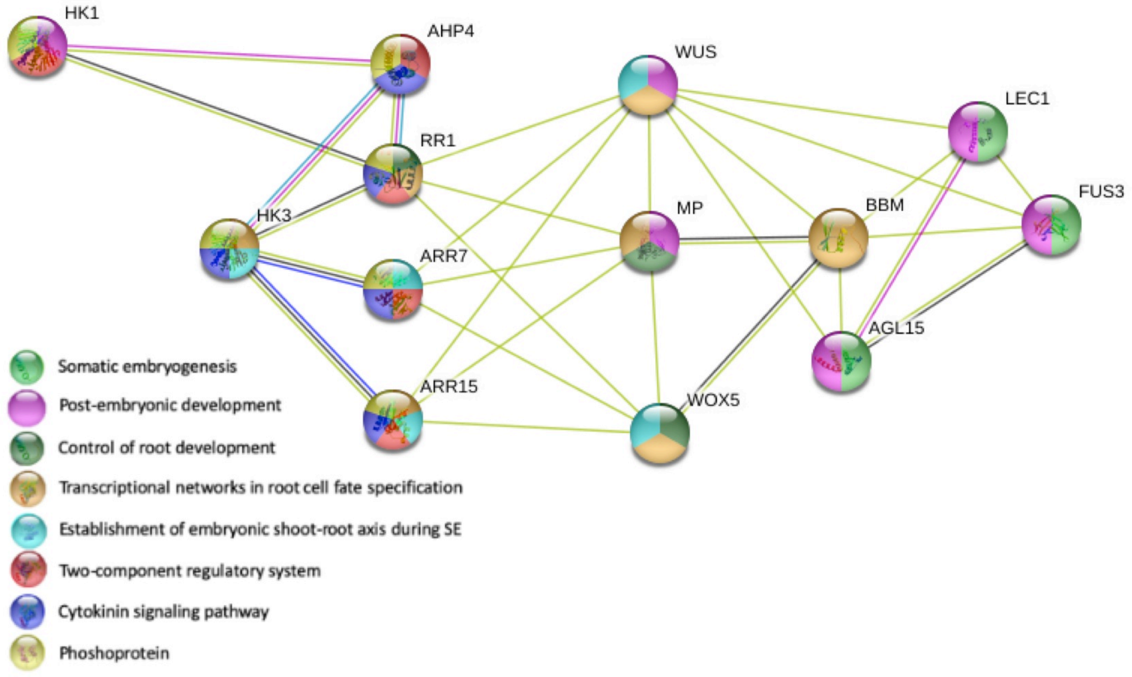

Supplementary Figure 2. Gene network using STRING database with high confidence (0.700) of two component and cytokinin signaling genes, AHK1, AHK3, AHP4, ARR1, ARR7, ARR15, Auxin signaling; ARF5 (Monopteros), homedomains and master regulators of SE.

Osmotic stress-induced somatic embryo maturation of coffee Coffea arabica L, shoot and root apical meristems development and robustness.

Eliana Valencia-Lozano[1], Jorge E. Ibarra[1], Humberto Herrera-Ubaldo[3], Stefan De Folter[3], José L. Cabrera-Ponce[2*]

Supplementary Table 1. Sequence primer qPCR and protein function of genes analyzed during SEs in *C. arabica* var. Typica.

**Gene**	**ID AT (*A. thaliana*)**	**ID XP (*C. arabica*)**	**Protein function**	**% ID At/Ca**	Primer
AHK1	AT2G17820.1	XP_027075540	Histidine kinase 1; Functions as an osmosensor histidine kinase that detects water stress and transmits the stress signal to a downstream MAPK cascade.	64.07	5´ CTTAGCAGCGGGAAGTGTTC 3´<br>3´ CCATGCAAGCGAGTAGTTGA 5´
AHK3	AT1G27320.1	XP_027097259	Histidine kinase 3; Cytokinins (CK) receptor related to bacterial two-component regulators.	67.12	5´ TGGAAGATGGAGAGGATTGG 3´<br>3´ ATTCCCACGATCTTCCTGTG 5´
AHP4	AT3G16360	XP_027125278	*Arabidopsis thaliana* histidine phosphotransfer proteins (AHPs). Encodes AHP4, a histidine-containing phospho-transmitter involved in Histidine (His)-to-Aspartate (Asp) phosphorelay signal transduction. AHP4 is one of the six	70.34	5´ TCAACTGCGACGAAAACTTG 3´<br>3´ GGTTCTTCTCCAGTGCTTGC 5´
ARR1	AT3G16857.2	XP_027115945	Two-component response regulator ARR1; Transcriptional activator that response regulator involved in His-to-Asp phosphorelay signal transduction system.	52.11	5´ TTGCCCGGTAATAGTTTTGC 3´<br>3´ CATTTTGCATACCCCAATCC 5´
ARR7	AT1G19050.1	XP_027098055.1	Arabidopsis response regulator (ARR) family, most closely related to ARR15. A two-component response regulator protein containing a phosphate accepting domain in the receiver domain but lacking a DNA binding domain in the output domain.	63.35	5´AGGGTTTGGCAAGAGAAAATTG 3´<br>3´AGTTTGTGTTGGTGAGGAG 5´
ARR15	AT1G74890.1	XP_027065231.1	Two-component response regulator ARR15; Functions as response regulator involved in His-to-Asp phosphorelay signal transduction system. Phosphorylation of the Asp residue in the receiver domain activates the ability of the protein to promote the transcription of target genes.	50.85	5´ GTGGGTTGTGGATTTTGGTG 3´<br>3´GTATGAGCTGAGGTGAGATGAG 5´
WUS	AT2G17950.1	XP_027100463.1	Homeodomain-like superfamily protein; Transcription factor that plays a central role during early embryogenesis, oogenesis and flowering. Required to specify stem cell identity in meristems, such as shoot apical meristem (SAM).	40.4	5´ CCGGTCCCAGAATTAGAAACTC 3´<br>3´GAATAACCCACGTTGCCATTG 5´
WOX5	AT3G11260.1	XP_027109768.1	WUSCHEL related homeobox 5; Transcription factor, which may be involved in the specification and maintenance of the stem cells (QC cells) in the root apical meristem (RAM)	58.54	5´ TCGCAGAGTTTCGATTGATG 3´<br>3´ GCTGCTTCTCTGCCTCTGAT 5´
ARF5	AT1G19850.1	XP_027090708.1	Transcriptional factor B3 family protein / auxin-responsive factor AUX/IAA-related; Auxin response factors (ARFs) are transcriptional factors that bind specifically to the DNA sequence.	58.01	5´CAATTGAGCGGATGTTTGGAC 3´<br>3´TCCACATACACCAGTTTCCAG 5´
LEC1	AT1G21970.1	XP_027085797	Nuclear transcription factor Y subunit B-9; Transcriptional activator of genes required for both embryo maturation and cellular differentiation. Sequence is similar to HAP3.	76.47	5' CCAGGAATGTGTATCGGAGTAC 3´<br>3´GAAAGCGGTGGAGATATAGGG 5´
BBM	AT5G17430	XP_027062561	AP2-like ethylene-responsive transcription factor BBM; Transcription factor that promotes cell proliferation, differentiation and morphogenesis, especially during embryogenesis	41.53	5´ TTCAACCCCAACGAGATCAG 3´<br>3´GTTGTAGTTCTCCTTCCAGTCC 5´
FUS3	AT3g26790.1	XP_027102113.1	Transcriptional factor with high similarity to the B3 region of the VP1/ABI3-like proteins. Present in many seed-specific promoters.	44.92	5´GGCTTACGACATGGAGACTAC 3´<br>3´GCATTTATCTCCGACTCAGGG 5´
AGL15	AT5G13790.1	XP_027113896.1	AGL15 (AGAMOUS-Like 15) is a member of the MADS domain family of regulatory factors. Is preferentially expressed during embryogenesis, may play a role during post-germinative development.	55.77	5´ TGGGAGGAAGAATTCTGGTG 3´<br>3´CTAACAGGCAAACACGCTGA 5´

Osmotic stress-induced somatic embryo maturation of coffee Coffea arabica L, shoot and root apical meristems development and robustness.

Eliana Valencia-Lozano[1], Jorge E. Ibarra[1], Humberto Herrera-Ubaldo[3], Stefan De Folter[3], José L. Cabrera-Ponce[2*]

Supplementary Table 2. Genes involved in SEs maturation of *C. arabica* identified from differentially expressed genes (DEGs).

Non-osmotic stress M3	osmotic stress M9	GENE ID	Annotation in the *Coffea arabica* genome
0.25	2.29	AHK1	XP_027075540
1.12	-0.27	AHK3	XP_027097259
-1.47	1.94	AHP4	XP_027125278
-3.84	4.22	ARR1	XP_027115945
1.32	-3.13	ARR7	XP_027098055.1
0.68	-2.69	ARR15	XP_027065231.1
-2	1.89	ARF5	XP_027090708.1
-5.06	3.53	WUS	XP_027100463.1
-0.79	5.06	WOX5	XP_027109768.1
-6.64	5.81	LEC1	XP_027085797
-5.64	4.27	FUS3	XP_027102113.1
-5.06	4.36	BBM	XP_027062561
0.95	2.15	AGL15	XP_027113896.1

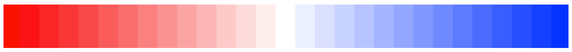

-7 0 7

*SE-M3: Embryogenic medium with 3 g/L gelrite; SE.M9: Embryogenic medium with 9 g/L gelrite. See text and Table S1 for the identification of genes.
