# Supplementary figures and images for: Osmotic stress-induced somatic embryo maturation of coffee Coffea arabica L., shoot and root apical meristems development and robustness
Source: Sci Rep. 2021 May 6;11:9661. doi: 10.1038/s41598-021-88834-z (PMC8102543; doi:10.1038/s41598-021-88834-z)

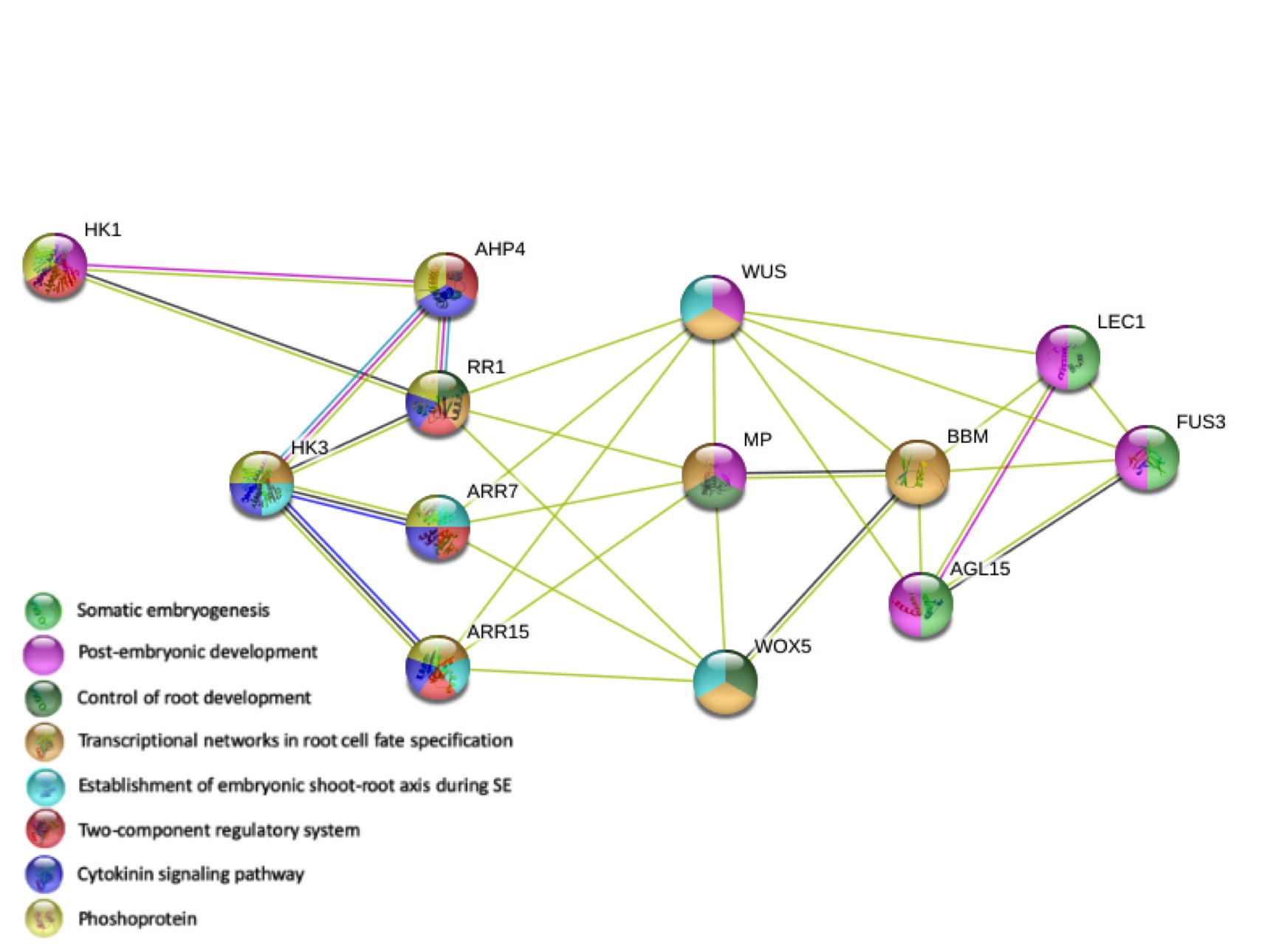

Supplement: Supplementary file 4 — Supplementary Information 4. [file 41598_2021_88834_MOESM4_ESM.jpg]
